# Case report: Efficacy of tofacitinib in the treatment of generalized pustular psoriasis

**DOI:** 10.3389/fmed.2025.1518583

**Published:** 2025-02-17

**Authors:** Xian Wang, Mei Zhang, Ming He, Ting Tang

**Affiliations:** ^1^Guizhou University of Traditional Chinese Medicine, Guiyang, China; ^2^Department of Dermatology, First Affiliated Hospital of Guizhou University of Traditional Chinese Medicine, Guiyang, China

**Keywords:** generalized pustular psoriasis, Tofacitinib, JAK inhibitors, case report, psoriasis

## Abstract

Generalized pustular psoriasis (GPP) is a rare and potentially life-threatening autoimmune inflammatory skin disease. Tofacitinib is a non-selective, first-generation Janus kinase (JAK) inhibitor. Currently, both domestic and international reports regarding the use of tofacitinib mainly have primarily focused on the treatment of psoriasis vulgaris and psoriatic arthritis. As GPP is a rare skin disease, reports on the use of tofacitinib in the treatment of GPP are rare. This report presents a case of severe GPP that was effectively treated with tofacitinib. This case suggests that this non-selective JAK inhibitor, which has strong anti-inflammatory effects, could serve as an effective treatment option for cases of acute exacerbation of GPP, with a good safety profile.

## 1 Introduction

**Figure 1:**
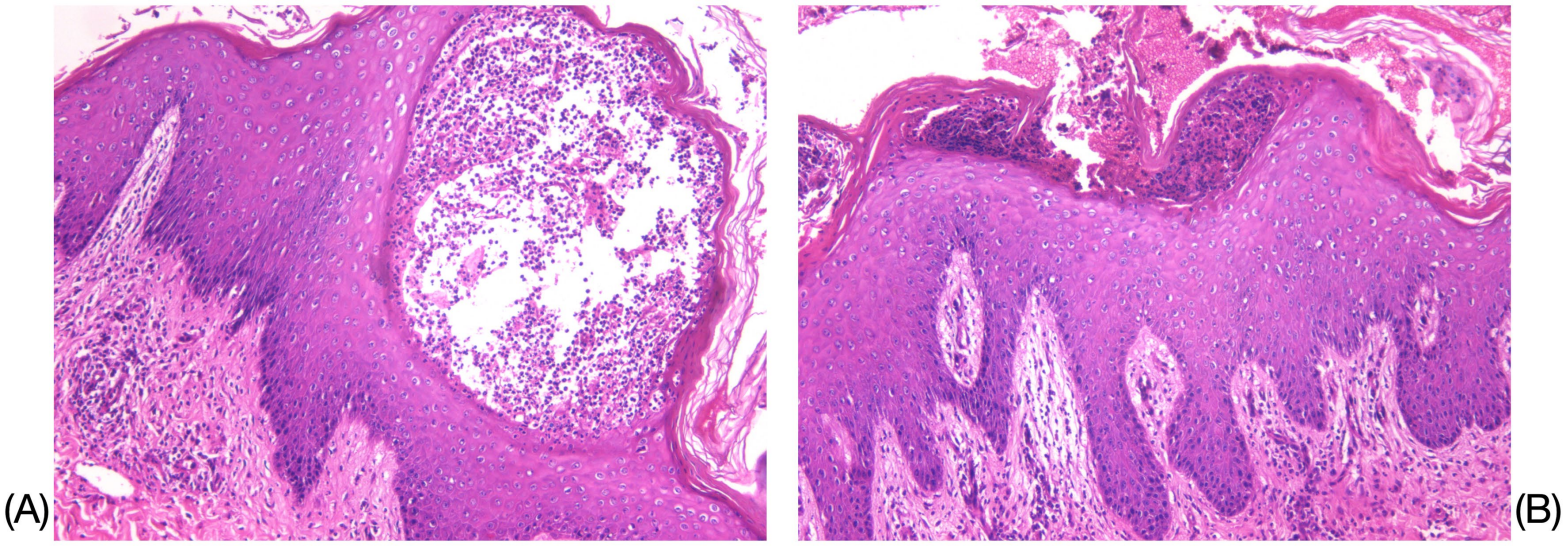
Histopathological results of the left upper-extremity skin lesion. **(A)** Mild hyperkeratosis with parakeratosis in the epidermis was observed, accompanied by Munro’s microabscess-like changes and pustule formation within the epidermis (HE×200). **(B)** Localized neutrophilic infiltrates were observed within the stratum corneum and stratum granulosum, while spiny layer cells exhibited hyperplasia and spongiotic edema (HE×100).

**Figure 2:**
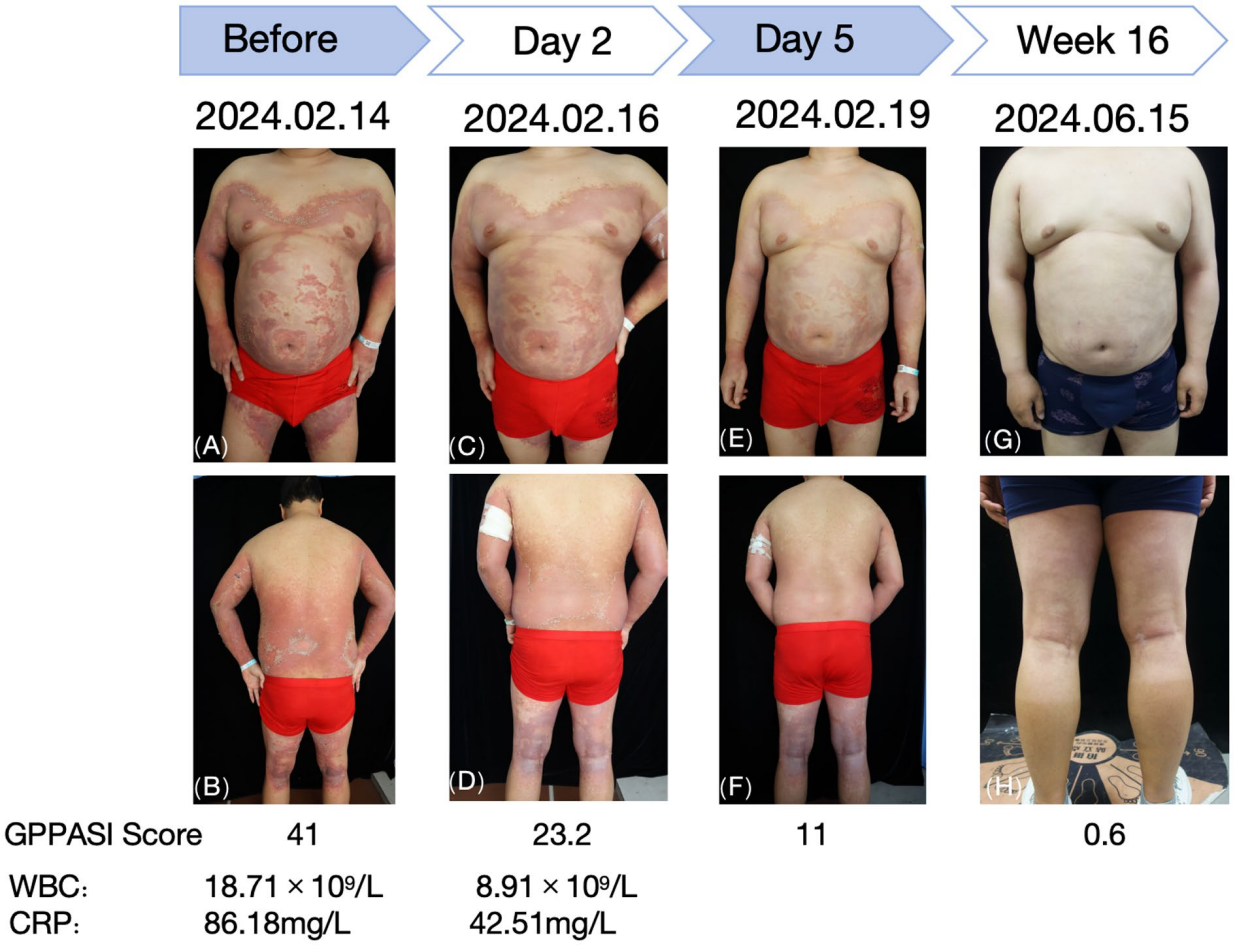
Changes observed in the skin lesions before and after treatment with tofacitinib. **(A,B)** Before treatment. **(C,D)** Day 2 of treatment. **(E,F)** Day 5 of treatment. **(G,H)** Week 16 of treatment.

**Figure 3:**
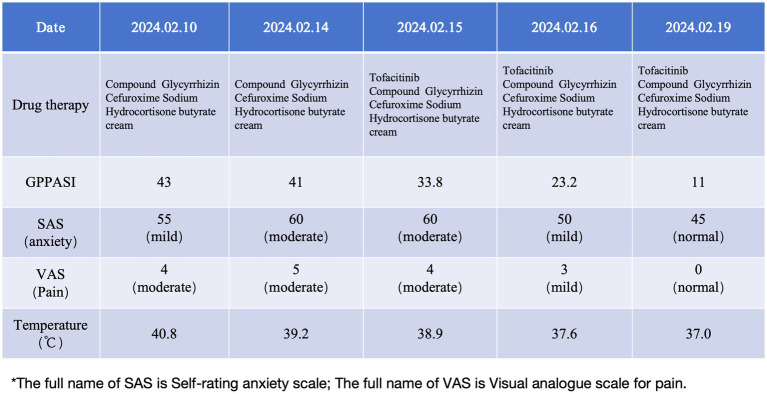
Timeline of the episode of care.

Generalized pustular psoriasis (GPP) is a rare and potentially life-threatening autoinflammatory skin condition. Its treatment poses challenges owing to the limited number of therapeutic options, with existing treatments lacking satisfactory efficacy and safety. Increasing evidence suggests that the pathogenesis of GPP is associated with numerous genetic mutations. Mutations in the *IL36RN* gene lead to a deficiency in the interleukin-36 receptor antagonist (IL-36Ra), which is the primary contributing factor in GPP (1–4). IL-36R, via the NF-KB signaling pathway and mitogen-activated protein kinases (MAPKs), induces the production of pro-inflammatory cytokines and chemokines (such as IL-1, IL-6, IL-8, and IL-36). Concurrently, dendritic cells (DCs) secrete TNF-*α* and IL-23, thereby activating neighboring Th17 cells. The activated Th17 cells produce IL-17A, which enhances the synthesis of additional IL-36R agonists, such as IL-36a, IL-36B, and IL-36γ, thereby further exacerbating the resulting cytokine storm. This massive cytokine storm activates various inflammatory mediators and attracts abundant neutrophils to the epidermis, which, in turn, leads to the pustular rash characteristic of GPP. Respiratory infections, which can lead to immune dysregulation, are among the triggering factors of GPP and may be related to GPP recurrence (5). In this report, we present a case of GPP treated with tofacitinib, which showed significant improvement after 5 days of treatment. Tofacitinib is known for its strong anti-inflammatory effects; therefore, we explored its therapeutic potential in this severe case of GPP (Figures 1–3).

## 2 Case description

A 28-year-old male patient presented with recurrent generalized erythema, pustules, and scales spread over the entire body, which had persisted for more than 4 years. He was diagnosed with GPP at an external institution 4 years earlier, where he was treated with oral acitretin (30 mg/qd), methotrexate (10 mg/qw), and an unspecified traditional Chinese medicine (TCM) taken orally, in addition to TCM creams and glucocorticoid ointments applied topically. Although these treatments initially improved his symptoms, the patient subsequently too acitretin or methotrexate only irregularly, and the skin rashes have repeatedly recurred. The aforementioned oral medications were discontinued 6 months ago. The patient denied any family history of this disease. Around 3 days before admission, a recurrence was triggered by an upper respiratory infection, manifesting as erythema, pustules, and scales on the limbs, trunk, and head, which progressively spread across the entire body. The patient reported experiencing systemic skin pain and a fever with an unspecified peak temperature. The self-administration of unspecified TCM creams at home provided inadequate relief. Upon examination, the patient’s vital signs were recorded as follows: temperature at 40.8°C; respiration rate of 19 breaths/min; and heart rate of 106 beats/min. The posterior pharyngeal wall was congested with grade II tonsillar enlargement without suppuration. No other significant abnormalities were detected during the systemic examination. The dermatological examination revealed generalized erythema and dryness, along with large swollen erythematous patches that were covered with densely packed, pinhead-to-millet-sized, pale yellow or yellowish superficial pustules. Some pustules fused to form pustular lakes, surrounded by fine scaly flakes resembling dandruff and collar-like areas of exfoliation, which elicited tenderness upon palpation. These manifestations were most prominent on the chest, back, axillae, buttocks, and popliteal fossa. The physician’s overall assessment based on the Generalized Pustular Psoriasis Physician Global Assessment (GPPGA) score was 4, while the Generalized Pustular Psoriasis Area and Severity Index (GPPASI) score was 41. The laboratory test results indicated an elevated white blood cell (WBC) count of 18.71 × 10^9^/L, C-reactive protein (CRP) levels of 86.18 mg/L, and an erythrocyte sedimentation rate (ESR) of 92.00 mm/h. Liver and kidney function tests, electrolyte levels, throat swabs, blood cultures, and T-spot test results revealed no significant abnormalities. The histopathological examination of the skin lesions (left upper extremity) revealed mild hyperkeratosis with parakeratosis in the epidermis, accompanied by Munro’s microabscess-like changes and pustule formation within the epidermis. Localized neutrophilic infiltrates were observed within the stratum corneum and stratum granulosum. The spiny layer cells exhibited hyperplasia and spongiotic edema. Mild edema was present in the superficial dermis, while inflammatory cell infiltration was noted around the subcutaneous tissue and small blood vessels, which was consistent with the pathological changes typically seen in pustular psoriasis.

## 3 Diagnostic assessment

The patient was intravenously administered compound glycyrrhizin and cefuroxime sodium, while hydrocortisone butyrate cream and skin emollients were prescribed for external use. As the patient reported poor efficacy with the previous treatment regimen and noted the unpleasant dryness and teratogenic effects of acitretin, he declined the use of acitretin and immunosuppressants. Moreover, due to financial constraints, biological agents (IL-36R monoclonal antibody—spesolimab) were refused during hospitalization. After a thorough discussion and obtaining informed consent, the patient was prescribed tofacitinib citrate tablets at a dosage of 5 mg twice daily. After 3 days of treatment, the erythema began to diminish and the pustules started to dry. By day 5, the erythema had markedly decreased and the pustules had largely resolved, with the GPPGA score dropping to 1 and the GPPASI score dropping to 11. Follow-up blood tests revealed the following results: WBC: 8.91 × 10^9^/L, CRP: 42.51 mg/L, ESR: 120.00 mm/h, IL-6 < 1.50 pg./mL, IL-8: 33.40 pg./mL, IL-10 < 5 pg./mL, and TNF-*α*: 18.7 pg./mL.

After 2 weeks, the erythema notably decreased, and the dosage of tofacitinib citrate tablets was reduced to 5 mg once daily. This dose has been maintained regularly to date, without adding any other oral medications. The regular blood tests and evaluations of the liver and kidney function throughout this period revealed no significant abnormalities. At the 16-week follow-up, there was no recurrence of skin lesions, and the patient reported no unusual discomfort.

## 4 Discussion

Tofacitinib is a first-generation, non-selective Janus kinase (JAK) inhibitor that simultaneously binds to the homologous targets JAK1, JAK2, JAK3, and TYK2, thereby preventing the activation of the JAK–STAT signaling pathway and reducing the production of various pro-inflammatory factors, such as interleukins IL-6, IL-8, IL-22, and INF-*γ* (6). In GPP lesions, the proliferation of epidermal keratinocytes (KCs) leads to a significant increase in IL-36 agonist expression. Morelli et al. (7) demonstrated that tofacitinib not only directly acts on the JAK-dependent signaling activated by TNF-*α*-induced cytokines (e.g., IL-6), which in turn activates STAT3 through an autocrine loop, but also inhibits IL-22 and INF-*γ* via the JAK1/TYK2–STAT and JAK1/2–STAT1 pathways, thereby suppressing the proliferation and differentiation of KCs. Animal studies conducted by Calama et al. (8) confirmed that tofacitinib reduces inflammation by inhibiting neutrophil production through the JAK/STAT3 signaling pathway. Monocyte-derived dendritic cells (Mo-DCs) play a pivotal role in inflammation and infection, and the JAK/STAT pathway is one of the key pathways involved in Mo-DC differentiation. Marzaioli et al. (9), in one *in vitro* study, reported that tofacitinib inhibited the differentiation and function of Mo-DCs by modulating the balance of NADPH oxidase, thereby reducing the secretion of pro-inflammatory factors. These pro-inflammatory factors are involved in the pathogenesis of GPP, and their blockade mitigates inflammation and damage caused by the activation of inflammation-induced immune system dysregulation, ultimately achieving therapeutic goals.

In 2012, tofacitinib was approved by the US Food and Drug Administration (FDA) for the treatment of moderate-to-severe rheumatoid arthritis (RA). Subsequently, it was approved for several other conditions, including psoriatic arthritis and ulcerative colitis, and it is currently a commonly used drug for treating rheumatic diseases (10). In the pathogenesis of GPP, tofacitinib can inhibit various inflammatory factors, including IL-1, IL-6, IL-17, and IL-23, via inhibition of the JAK–STAT pathway. It thus exerts a powerful anti-inflammatory effect. Compared to traditional drugs (such as acitretin, cyclosporine, and methotrexate), tofacitinib can achieve curative effects more rapidly. Although this drug has been associated with potential dose-dependent adverse reactions (such as severe infections, herpes zoster infections, malignancies, and thrombosis) in multiple systematic reviews and meta-analyses, the incidence of adverse events in patients receiving tofacitinib treatment did not increase, except for an elevated risk of herpes zoster infection (11, 12). These results highlight the importance of considering the safety data of JAK inhibitors in the context of the potential risks observed in different disease populations. Compared to the adverse reactions of traditional oral drugs such as acitretin, which can cause uncomfortable dryness, teratogenic effects, liver function impairment, and elevated blood lipid levels, and oral cyclosporine, which can cause increased blood pressure and gingival hyperplasia, tofacitinib is associated with a lower incidence of adverse reactions. In terms of health economics, the relatively low price of tofacitinib offers a specific advantage over traditional drugs and biologics, especially for less developed countries or regions, as well as populations with a heavy economic burden, in which the low cost of drugs remains a primary factor for consideration. To reduce the occurrence of adverse events, dermatologists should comprehensively assess each patient’s condition. Before administering the drug, absolute contraindications—such as tuberculosis, hepatitis B, tumors, and thrombosis—should be excluded. During treatment, concurrent use of immunosuppressants, such as methotrexate or corticosteroids, should also be avoided. After starting the drug, relevant indicators should be regularly monitored to detect any potential adverse reactions at an early stage. Regarding dosage, the amount used for plaque psoriasis and rheumatoid arthritis should be considered. For safety reasons, the recommended dosage for adults is 5 mg/bid.

Unfortunately, tofacitinib has several limitations as a drug treatment for GPP. First, there are rare clinical cases involving this drug in the treatment of GPP, and higher-level evidence from evidence-based medicine is also lacking. Furthermore, there are certain restrictions on the applicable population, such as older adults and those with potential thromboembolic risks, malignancies, and infections. Therefore, strict screening is required before medication administration to minimize the occurrence of serious adverse reactions. Compared to targeted biologic agents (such as lxekizumab and spesolimab), tofacitinib may have a slower onset of action.

Currently, tofacitinib is predominantly used, both nationally and internationally, to treat plaque psoriasis and psoriatic arthritis (13). However, because GPP is a rare skin disease, there is limited evidence regarding the use of tofacitinib in GPP treatment, due to the rarity of reports (14). JAK inhibitors have further been shown to effectively treat localized pustulosis. For example, upadacitinib has successfully treated refractory palmoplantar pustulosis with good safety (15). Given the overlapping pathogenesis of GPP and plaque psoriasis, traditional drug therapies often yield unsatisfactory results, and biological agents, such as ixekizumab and secukinumab, exhibit varying efficacy levels in patients with GPP. The first IL-36R antagonist, spesolimab, has been approved by the National Medical Products Administration (NMPA) for GPP treatment (16); however, its limited accessibility in primary hospitals due to high costs necessitates the urgent exploration of other safe and effective treatment options.

## 5 Conclusion

This report presents a case of severe GPP that was effectively treated with tofacitinib. The patient showed significant improvement after 5 days of tofacitinib therapy. Although pre-treatment inflammatory markers (such as IL-6 and L-8) were not comprehensively evaluated, which limited a complete assessment of the severity of inflammation prior to treatment, the patient’s clinical symptoms, signs, and post-treatment inflammatory marker levels indicated a notable suppression of the inflammatory response. Subsequently, the skin rash significantly subsided, with no recurrence observed during the 16-week follow-up, and no adverse clinical effects were reported. Regular blood tests and assessments of the liver and kidney function indicated a good safety profile. This case highlights the potent anti-inflammatory effects of the non-selective JAK inhibitor tofacitinib that may serve as an effective treatment option for patients with acute episodes of GPP and has a favorable safety profile. During treatment, patients should be advised to minimize exposure to or contact with other patients to prevent infection. Future research should prioritize conducting more foundational studies to assess the dosage, efficacy, treatment duration, and safety of tofacitinib in managing GPP.

## Data Availability

The original contributions presented in the study are included in the article/supplementary material, further inquiries can be directed to the corresponding authors.
